# Cytogenomic identification and long-read single molecule real-time (SMRT) sequencing of a *Bardet–Biedl Syndrome 9* (*BBS9*) deletion

**DOI:** 10.1038/s41525-017-0042-3

**Published:** 2018-01-22

**Authors:** Jennifer Reiner, Laura Pisani, Wanqiong Qiao, Ram Singh, Yao Yang, Lisong Shi, Wahab A. Khan, Robert Sebra, Ninette Cohen, Arvind Babu, Lisa Edelmann, Ethylin Wang Jabs, Stuart A. Scott

**Affiliations:** 1 Department of Genetics and Genomic Sciences, Icahn School of Medicine at Mount Sinai, New York, NY 10029 USA; 2 Sema4, a Mount Sinai Venture, Stamford, CT 06902 USA; 3 Icahn Institute for Genomics and Multiscale Biology, Icahn School of Medicine at Mount Sinai, New York, NY 10029 USA; 4 Present Address: Sanford Genetics and Genomic Laboratory, Sioux Falls, SD 57105 USA; 5 Present Address: Department of Pediatrics, Division of Medical Genetics, Northwell Health, Lake Success, NY 11020 USA; 6 Present Address: Division of Cytogenetics and Molecular Pathology, Donald and Barbara Zucker School of Medicine at Hofstra Northwell, Northwell Health Laboratories, Lake Success, NY 11042 USA

## Abstract

Bardet–Biedl syndrome (BBS) is a recessive disorder characterized by heterogeneous clinical manifestations, including truncal obesity, rod-cone dystrophy, renal anomalies, postaxial polydactyly, and variable developmental delays. At least 20 genes have been implicated in BBS, and all are involved in primary cilia function. We report a 1-year-old male child from Guyana with obesity, postaxial polydactyly on his right foot, hypotonia, ophthalmologic abnormalities, and developmental delay, which together indicated a clinical diagnosis of BBS. Clinical chromosomal microarray (CMA) testing and high-throughput BBS gene panel sequencing detected a homozygous 7p14.3 deletion of exons 1–4 of *BBS9* that was encompassed by a 17.5 Mb region of homozygosity at chromosome 7p14.2–p21.1. The precise breakpoints of the deletion were delineated to a 72.8 kb region in the proband and carrier parents by third-generation long-read single molecule real-time (SMRT) sequencing (Pacific Biosciences), which suggested non-homologous end joining as a likely mechanism of formation. Long-read SMRT sequencing of the deletion breakpoints also determined that the aberration included the neighboring *RP9* gene implicated in retinitis pigmentosa; however, the clinical significance of this was considered uncertain given the paucity of reported cases with unambiguous *RP9* mutations. Taken together, our study characterized a *BBS9* deletion, and the identification of this shared haplotype in the parents suggests that this pathogenic aberration may be a BBS founder mutation in the Guyanese population. Importantly, this informative case also highlights the utility of long-read SMRT sequencing to map nucleotide breakpoints of clinically relevant structural variants.

## Introduction

Bardet–Biedl syndrome (BBS) is a rare genetic disorder characterized by rod-cone dystrophy, truncal obesity, postaxial polydactyly, hypogonadism/genital anomalies, variable intellectual disability, and renal abnormalities. Secondary features can include hepatic fibrosis, diabetes mellitus, cognitive deficits, facial dysmorphism, short stature, and other pleotropic congenital abnormalities.^1,2^ Variable expression of primary and secondary features is common, which is due, in part, to locus heterogeneity, yet intrafamilial clinical variability is also observed in some BBS cases.^3^ The mode of BBS inheritance is predominantly autosomal recessive; however, a multi-allelic inheritance pattern has been suggested for some BBS families.^4,5^

A clinical diagnosis of BBS is based on the presence of primary and secondary clinical features;^6^ however, the recent availability of multi-gene sequencing panels has facilitated molecular diagnosis, estimation of recurrence risk, and targeted mutation testing for extended family members. To date, 20 genes involved in multi-subunit cilia assembly and function have been implicated in BBS,^7^ and the spectrum of pathogenic mutations in these genes includes loss-of-function missense, nonsense, and structural variants. Herein, we report a Guyanese proband with a homozygous *BBS9* deletion encompassed by a 17.5 Mb region of homozygosity on chromosome 7p14.2–p21.1. The identical heterozygous *BBS9* deletions and shared haplotypes in the parents suggests that this pathogenic aberration may be a founder mutation in the Guyanese population.

## Results

### Postnatal clinical evaluation

**Fig. 1:**
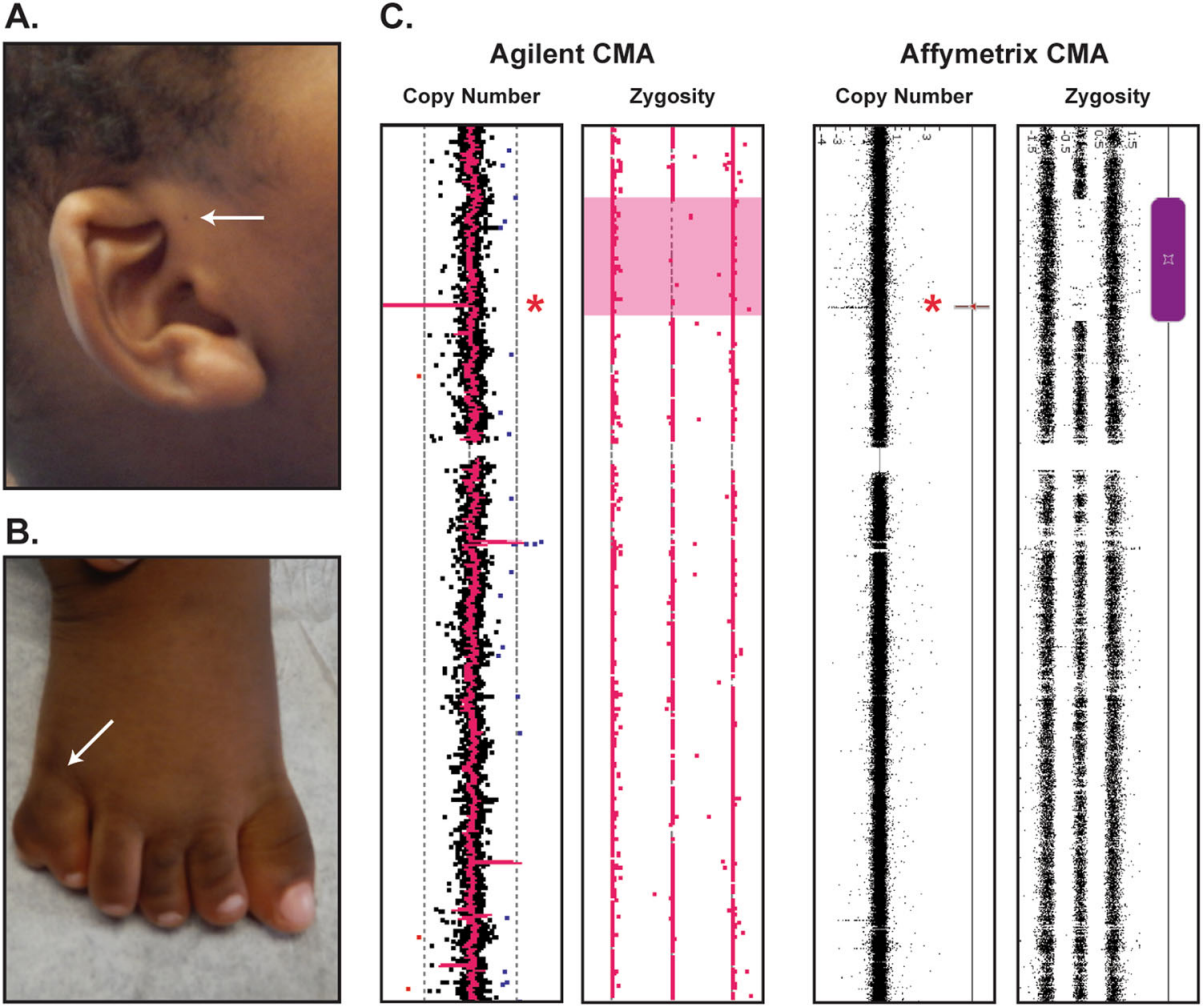
Clinical and genetic diagnosis of Bardet–Biedl syndrome. Clinical evaluation of a 14-month-old proband revealed features suggestive of BBS, including low-set ears and a right ear pit (arrow) (**a**), and postaxial polydactyly on the right foot (arrow) (**b**). Analysis of the patient specimen on the Agilent 4 × 180 CGH + SNP array (**c**, left panel) detected a single region of homozygosity at chromosome 7p14.2–p21.1. A homozygous deletion (indicated by an asterisk) of the 5′ region of *BBS9* was nested within the region of homozygosity at 7p14.3. Analysis of the proband DNA with the higher-resolution Affymetrix CytoScan HD array (**c**, right panel) confirmed these results and further refined the deletion breakpoints

At 12 months of age, the male proband had a history of excessive weight gain despite no reported excess in feeding, prompting a referral to Medical Genetics for evaluation of an overgrowth syndrome. He was the second child of healthy, reportedly non-consanguineous Guyanese parents. At 14 months, his weight was 19.16 kg (100th pct, +5.8 SD), his length was 84.4 cm (99th pct, +2.29 SD), his head circumference was 52 cm (100th pct, +4.06 SD), and his BMI was 26.9 kg/m^2^. Examination revealed a round face, low-set ears with fleshy lobes, a right ear pit (Fig. 1), a 2 × 1 cm café-au-lait spot on the right chest, and postaxial polydactyly on his right foot (Fig. 1). His genitalia appeared normal, partially hidden in a large fat pad, with a stretched penile length of 3.7 cm (25–50th pct). He demonstrated marked axial and appendicular hypotonia, and had significant difficulties in visual tracking. He started sitting up at 10 months but was unable to pull up to stand at 14 months. Although he could babble, he had not yet said his first word. His daily dietary intake consisted mostly of fruits, vegetables, and 12 ounces of breast milk, as carbohydrates were restricted in an attempt to limit weight gain. His comprehensive metabolic panels, including glucose and endocrine workup (TSH, T4-free thyroxine, T4 total, T3 total, IGF, and IGFBP-3), were normal. At 2 years, his cholesterol (214 mg/dl) and LDL (157 mg/dl) were elevated with his highest hemoglobin A1C at 5.7%. At 3 years, his vitamin D, 25-OH was low at 26 ng/ml. Kidney ultrasonography was normal at the time of evaluation; however, a 1-year follow-up was recommended to monitor for renal cysts and calyceal diverticula, which are common features among young children with BBS.^8,9^ Ophthalmologic examination showed small amplitude vertical nystagmus in association with reduced photopic and scotopic ERGs and normal fundi. Echocardiogram revealed a structurally normal heart with normal biventricular function. Auditory brain response revealed slight hearing loss at 500 Hz with normalization at higher frequencies. Based on these findings, a clinical diagnosis of BBS was made according to established criteria.^6^

### Clinical cytogenomic and molecular genetic analyses

Cytogenetic analysis of the proband revealed a normal male karyotype in peripheral blood lymphocytes; however, chromosomal microarray (CMA) testing detected a homozygous 7p14.3 deletion (minimum size: 43.0 kb; maximum size: 119.6 kb) nested within a 17.5 Mb region of homozygosity at chromosome 7p14.2–p21.1 (Fig. 1). The molecular karyotype was reported as: arr[hg19] 7p21.1p14.2(18969411_36497446)×2 hmz,7p14.3(33150658_33193639)×0. The minimum-sized deletion included exons 1–3 of *BBS9*, which is a known Mendelian disease gene implicated in autosomal recessive BBS. The homozygous *BBS9* deletion was independently confirmed by a clinical ciliopathy sequencing panel, which reported a homozygous loss of exons 2–4 of *BBS9*. Exon 1 of *BBS9* was not amenable to copy number analysis by sequencing due to the high GC content of this 5′ exon.

Analysis of parental DNA by CMA testing indicated that both parents were heterozygous carriers of the identical 7p14.3 deletion. The absence of additional regions of homozygosity throughout the proband’s genome supported the denial of known consanguinity.

### Deletion breakpoint identification

**Fig. 2:**
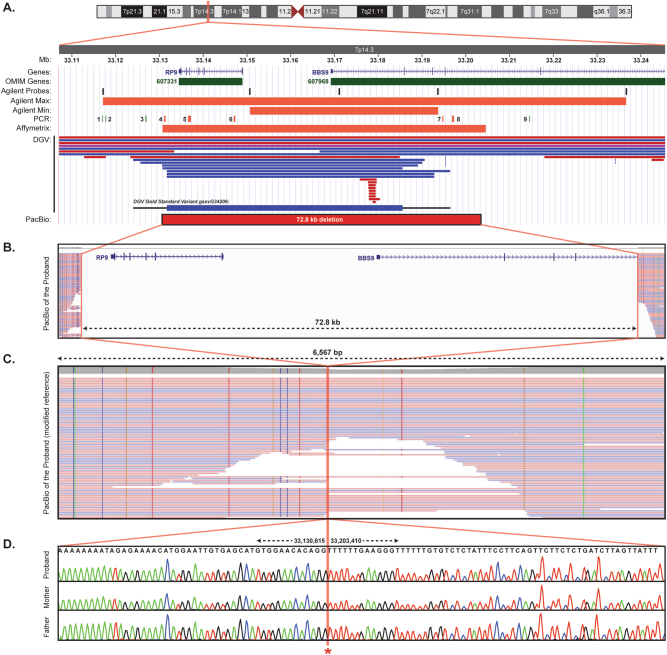
Deletion breakpoint identification. The chromosome 7p14.3 genomic region is illustrated with tracks for the proband chromosomal microarray (CMA) results, genomic PCR mapping amplicon locations (1–9; green: amplified; red: did not amplify), and copy number variants detected among healthy individuals in the Database of Genomic Variants (DGV; blue: duplication; red: deletion) (**a**). Unambiguous breakpoint mapping was performed by long-read single molecule real-time (SMRT) sequencing (PacBio) of long-range PCR products that amplified across the deleted interval in the proband (**b**). These SMRT sequencing data were also aligned to a modified human genome reference that excluded the identified 72.8 kb deletion (chr7:33130616–33203409) (**c**), confirming that there were no other sequence alterations at the breakpoint locations. The precise deletion breakpoints were subsequently confirmed in the proband and both carrier parents by Sanger sequencing of the long-range PCR amplicons (**d**)

Based on the probe spacing of the Agilent CMA platform, the identified homozygous deletion potentially included the neighboring retinitis pigmentosa gene, *RP9* (Fig. 2),^10^ which prompted additional studies to refine the deletion breakpoints to determine the risk of this condition in the proband and his carrier parents. As such, a series of PCR primers were designed to amplify specific regions between the minimum and maximum CMA deletion breakpoints. As illustrated in Fig. 2, only primer sets 1, 2, 3, and 9 successfully amplified in the proband, indicating that primer sets 4–8 were within the homozygous deletion. These results suggested that the entire *RP9* gene was included within the deletion, in addition to exons 1–4 of *BBS9*, and narrowed the distal and proximal deletion breakpoints.

Higher-resolution Affymetrix CMA testing of the proband further refined the deletion breakpoints (Fig. 2). Fine mapping was subsequently accomplished by long-range PCR across the narrowed breakpoints and subjecting the overlapping ~5.3–6.6 kb amplicons to third-generation single molecule real-time (SMRT) sequencing. Long-read SMRT sequencing, and confirmatory Sanger sequencing, identified the precise nucleotide breakpoints in the proband and heterozygous parents, which encompassed 72.8 kb of chr7:33,130,616–33,203,409 (hg19) (Fig. 2). The flanking sequence of the breakpoints did not reveal any repetitive or homologous elements; however, interrogation of the Database of Genomic Variants (DGV; https://dgv.tcag.ca) revealed that the reciprocal duplication of this deletion (DGV Gold Standard Variant gssvG34206) is found in the general population at a frequency of 0.27% (Fig. 2).

## Discussion

The *BBS9* gene encodes PTHB1, which is an essential component of the BBSome cilia trafficking protein complex.^11^ Although the specific function of PTHB1 is currently unclear, knockdown of *BBS9* expression in zebrafish decreases cilia density and length, and causes brain and eye abnormalities that are rescued by ectopic expression of human *BBS9* mRNA.^12^ Since the initial *BBS9* mutation families were identified,^13^ it is estimated that *BBS9* accounts for ~6% of all BBS cases.^1,9^ The spectrum of reported *BBS9* mutations suggests that loss-of-function single-nucleotide variants (e.g., splicing, nonsense, and frameshifts) and larger gene-disrupting copy number variants (CNVs) are pathogenic. To date, deletions of exons 8–9, exon 9, and exon 31 of *BBS9* have been identified in probands with BBS.^14,15^ Our study extends the *BBS9* mutation landscape to now include a 72.8 kb deletion of exons 1–4 and the neighboring *RP9*.

Notably, ophthalmologic evaluation of the proband and his parents did not detect evidence of retinitis pigmentosa to date, suggesting that *RP9* variants may present with later onset, have incomplete penetrance, or that the *RP9* missense mutations (c.410A > T; p.His137Leu and c.509A > G; p.Asp170Gly) previously reported in a single family may actually be benign rare variants.^10^ Of these two reported variants, only c.509A > G (rs104894039) was observed in the Genome Aggregation Database (gnomAD; http://gnomad.broadinstitute.org/), but with a very rare minor allele frequency (0.000065). Of note, a frameshift *RP9* variant has also been reported among individuals with hereditary retinal dystrophy (c.664delT; p.Ter222Aspfs; rs553265417); however, its appreciable European (non-Finnish) allele frequency in gnomAD (0.01083) suggests that it is likely benign.^16^ Given the uncertain clinical significance of homozygous or heterozygous loss of *RP9*, continued ophthalmologic evaluation of the proband and parents is planned.

The 7p14.3 deletion breakpoints were identified by an innovative long-read SMRT sequencing strategy, which revealed a low-copy repeat/LTR and a LINE element at the distal and proximal breakpoints, respectively. The absence of sequence homology at both breakpoints excluded non-allelic homologous recombination and microhomology-mediated break-induced repair as mechanisms of CNV formation. However, analysis of the deleted region revealed several inverted repeats with homology ranging from 74 to 100%. Inverted repeats can form hairpins and cruciform structures, and the resultant fork-stalling and DNA breakage can lead to non-homologous end joining.^17^ As noted above, the DGV indicates that the reciprocal 7p14.3 duplication occurs in the general population (0.27% frequency), which supports the hypothesis that a predisposition for rearrangement exists at 7p14.3 due to local genomic architecture.

Interestingly, the homozygous 7p14.3 deletion was encompassed by a single 17.5 Mb region of homozygosity at 7p14.2–p21.1. Familial consanguinity was denied; however, both parents indicated they were from the small Mahaicony region of Guyana. Guyana is one of the smallest countries in mainland South America, but is ethnically diverse with its population being comprised of Indian, African, and Amerindian ancestry. Of note, Guyanese founder mutations have previously been reported, including a *LIPH* mutation on chromosome 3q27.2 that results in autosomal recessive woolly hair/hypotrichosis.^18^

In conclusion, we describe a homozygous *BBS9* deletion detected in a BBS proband from a Guyanese family, with precise breakpoints delineated by long-read SMRT sequencing. Long-read SMRT sequencing also determined that the deletion encompassed the neighboring retinitis pigmentosa gene, *RP9*; however, the clinical significance of this was considered uncertain. Importantly, the identification of this shared *BBS9* deletion haplotype in the parents suggests that further studies are warranted to determine the allele frequency and incidence of BBS in the Guyanese population.

## Methods

### Clinical evaluation

Clinical evaluation was performed at the Genetics Clinic at the Icahn School of Medicine at Mount Sinai by genetic counselors and clinical geneticists (L.P. and E.W.J.). The family provided written informed consent for Icahn School of Medicine at Mount Sinai Institutional Review Board-approved research participation and publication of photographs.

### Clinical cytogenetic and molecular genetic testing

Clinical cytogenomic testing included peripheral blood G-banded chromosome and CMA analysis performed at Mount Sinai Genomics Inc. (DBA Sema4; previously known as the Mount Sinai Genetic Testing Laboratory). DNA from the proband was tested on the SurePrint G3 ISCA CGH+SNP 4 × 180K array (Agilent Technologies, Santa Clara, CA), as per the manufacturer’s instructions and as previously described.^19,20^ Higher-resolution CMA testing of the proband on the CytoScan^®^ HD platform (Affymetrix, Santa Clara, CA) was performed per the manufacturer’s instructions and as previously reported.^20^

Clinical molecular testing included a 55-gene ciliopathy sequencing panel (Supplemental Table 1) performed at Invitae (San Francisco, CA). Enrichment and sequencing was restricted to coding regions and 10 bp of flanking intronic sequence at a minimum depth of 50×. Exonic deletions and duplications were assessed using a proprietary algorithm that compared read depth of target sequences in the proband to internal control samples.

### *BBS9* breakpoint identification

*Genomic DNA PCR amplification*: The deletion breakpoints were interrogated by PCR amplification of genomic DNA using a series of primers designed to generate amplicons at specific regions between the minimum and maximum deletion coordinates estimated by CMA testing (Supplemental Table 2).

*Long-read* *SMRT* *sequencing*: Long-range PCR amplification across the identified breakpoint regions was accomplished using primers targeted to unique DNA sequences flanking the approximated deletion coordinates, and these amplicons were subjected to SMRTbell library construction and long-read SMRT sequencing (Pacific Biosciences, Menlo Park, CA). Long-range PCR reactions were performed in 50 µl containing ~100 ng of DNA, 1× LA PCR buffer II (TaKaRa), 0.4 µM of barcoded forward and reverse primers (Supplemental Table 2), 0.4 mM dNTPs, 1 µL DMSO, and 2.5 units of TaKaRa LA Taq HS. Amplification consisted of an initial denaturation step at 95 °C for 5 min followed by 10 amplification cycles (95 °C for 30 s, 61 °C for 30 s, and 72 °C for 10 min), another 20 amplification cycles (95 °C for 30 s, 56 °C for 30 s, and 72 °C for 10 min), and a final extension at 72 °C for 15 min. All PCR amplicons were purified, quantified, pooled, sequenced (P6-C4 PacBio protocol), and analyzed as previously described.^21^

### Data availability

The long-read SMRT sequencing FASTQ data are available from the NCBI Sequence Read Archive (SRA) using the SRP125431 experiment identifier: https://www.ncbi.nlm.nih.gov/sra/SRP125431 [*accn*].

## Electronic supplementary material

Supplemental Data
