# Supplementary material for: Cytogenomic identification and long-read single molecule real-time (SMRT) sequencing of a Bardet–Biedl Syndrome 9 (BBS9) deletion
Source: NPJ Genom Med. 2018 Jan 22;3:3. doi: 10.1038/s41525-017-0042-3 (PMC5778042; doi:10.1038/s41525-017-0042-3)
Supplement: Supplementary file 1 — Supplemental Data [file 41525_2017_42_MOESM1_ESM.pdf]

Cytogenomic Identification and Long-read Single Molecule Real-Time (SMRT) Sequencing of a *Bardet-Biedl Syndrome 9* (*BBS9*) Deletion

Running Title: ***Long-read SMRT Sequencing of a* BBS9 *Deletion***

Jennifer Reiner[1*], Laura Pisani[1†], Wanqiong Qiao[1,3], Ram Singh[1,3], Yao Yang[1,2,3], Lisong Shi[1,3], Wahab A. Khan[1,3], Ninette Cohen[1**], Arvind Babu[1,3], Lisa Edelmann[1,3], Ethylin Wang Jabs[1‡], and Stuart A. Scott[1,3‡]

[1] Department of Genetics and Genomic Sciences, Icahn School of Medicine at Mount Sinai, New York, NY 10029.

[2] Icahn Institute for Genomics and Multiscale Biology, Icahn School of Medicine at Mount Sinai, New York, NY 10029.

[3] Sema4, a Mount Sinai venture, Stamford, CT 06902.

[*] Current affiliation: Sanford Genetics and Genomic Laboratory, Sioux Falls, SD 57105.

[†] Current affiliation: Department of Pediatrics, Division of Medical Genetics, Northwell Health, Lake Success, NY, 11020.

[**] Current affiliation: Division of Cytogenetics and Molecular Pathology, Donald and Barbara Zucker School of Medicine at Hofstra Northwell, Northwell Health Laboratories, Lake Success, NY 11042.

SUPPLEMENTAL DATA

[‡] **CORRESPONDENCE TO:**

Ethylin Wang Jabs, MD
Professor and Vice Chair
Department of Genetics and Genomic Sciences
Icahn School of Medicine at Mount Sinai
New York, NY 10029
Tel. 212-241-3504
Fax. 212-426-9065
E-mail: ethylin.jabs@mssm.edu

Stuart A. Scott, PhD
Associate Professor
Department of Genetics and Genomic Sciences
Icahn School of Medicine at Mount Sinai
New York, NY 10029
Tel. 212-241-3780
Fax. 212-241-0139
E-mail: stuart.scott@mssm.edu

SUPPLEMENTAL TABLE S1. Genes included in the clinical ciliopathy next-generation sequencing panel (Invitae; San Francisco, CA)

AHI1, ARL13B, ARL6, B9D1, B9D2, BBS1, BBS10, BBS12, BBS2, BBS4, BBS5, BBS7, BBS9, CC2D2A, CCDC39, CCDC40, CEP290, DNAAF1, DNAAF2, DNAH11, DNAH5, DNA11, DNA12, DNAL1, DYNC2H1, FOXH1, GDF1, GLIS2, IFT80, INPP5E, INVS, IQCB1, MKK5, MKS1, NEK8, NODAL, NPHP1, NPHP3, NPHP4, OFD1, PKD2, PKHD1, RPGRIP1L, RSPH4A, RSPH9, TCTN1, TCTN2, TMEM216, TMEM67, TRIM32, TTC21B, TTC8, WDPCP, XPNPEP3, ZIC3

SUPPLEMENTAL TABLE S2. Oligonucleotide primers for PCR amplification

Primer Sequence	T_a (cycles)	Product Length
Genomic DNA PCR Mapping:		
Amplicon 1<br>5'-ACTGAAGGATCCTCCCTTGG-3′<br>5'-CCATATGGGAGGTAAATCACAA-3′	60°C (30)	239 bp
Amplicon 2<br>5'-GGGAGTGTATTTCCCTTATCTTCA-3′<br>5'-CTGGGTTTGCCAAATTCTTC-3′	60°C (30)	232 bp
Amplicon 3<br>5'-AAAATGGCACCACTCTGGTT-3′<br>5'-GGAATTCCAGACTTGGGAGA-3′	60°C (30)	404 bp
Amplicon 4<br>5'-GGAGTCACAGAACAGCATTCC-3′<br>5'-TGAATGTTACCTGCTCCTGCT-3′	60°C (30)	348 bp
Amplicon 5<br>5'-CGGGAAATTAAATCCCTGAT-3′<br>5'-GATGCTTGGGGAAGCAATTA-3′	60°C (30)	614 bp
Amplicon 6<br>5'-TCTCATCTGTGAAAAAGCTCTCTG-3′<br>5'-ACCTTCTGGGAGGTTTGATG-3′	60°C (30)	343 bp
Amplicon 7<br>5'-TGTGCCATGCTAGTTTCTGG-3′<br>5'-TGTGAAACCATCTTGGAAAGG-3′	60°C (30)	205 bp
Amplicon 8<br>5'-TGAAAGAAACTGGCATATTAGGTG-3′<br>5'-GGGAGCAGTTGAAGAAAAGG-3′	60°C (30)	456 bp
Amplicon 9<br>5'-TTGGAGCTGCTCTTTCTGGT-3′<br>5'-GGCTTAAATTTGCCTGCTGA-3′	60°C (30)	191 bp

Long-range PCR:

Proband_3.1_8.2<br>5'-**TCAGACGATGCGTCAT**AATTTTTGGCTTGTGGGTGA-3′<br>5'-**AGTCATCGTATCGCGC**TCAGAACTTTAATGAACAGACTGACT-3′	61°C (10) / 56°C (20)	~6.6 kb
Proband_3.2_8.2<br>5'-**CATAGCGACTATCGTG**GGGTGATATCTAGAAATGGGAAG-3′<br>5'-**TGCATGCACAGATGCG**TCAGAACTTTAATGAACAGACTGACT-3′	61°C (10) / 56°C (20)	~5.3 kb
Mother_3.1_8.2<br>5'-**GTACACGCTGTGACTA**AATTTTTGGCTTGTGGGTGA-3′<br>5'-**TCTCTCACAGTCGAGC**TCAGAACTTTAATGAACAGACTGACT-3′	61°C (10) / 56°C (20)	~6.6 kb
Mother_3.2_8.2<br>5'-**TATCTCTGTAGAGTCT**GGGTGATATCTAGAAATGGGAAG-3′<br>5'-**TCTACAGAGCGAGAGT**TCAGAACTTTAATGAACAGACTGACT-3′	61°C (10) / 56°C (20)	~5.3 kb
Father_3.1_8.2<br>5'-**TCTATGTCTCAGTAGT**AATTTTTGGCTTGTGGGTGA-3′<br>5'-**TGTGATACTGCGAGCA**TCAGAACTTTAATGAACAGACTGACT-3′	61°C (10) / 56°C (20)	~6.6 kb
Father_3.2_8.2<br>5'-**CAGTGAGAGCGCGATA**GGGTGATATCTAGAAATGGGAAG-3′<br>5'-**ACAGACGCATATGTAC**TCAGAACTTTAATGAACAGACTGACT-3′	61°C (10) / 56°C (20)	~5.3 kb
Normal Control_3.1_8.2<br>5'-**TCAGACGATGCGTCAT**AATTTTTGGCTTGTGGGTGA-3′<br>5'-**AGTCATCGTATCGCGC**TCAGAACTTTAATGAACAGACTGACT-3′	61°C (10) / 56°C (20)	-
Normal Control_3.2_8.2<br>5'-**CATAGCGACTATCGTG**GGGTGATATCTAGAAATGGGAAG-3′<br>5'-**TGCATGCACAGATGCG**TCAGAACTTTAATGAACAGACTGACT-3′	61°C (10) / 56°C (20)	-

Nucleotides that are bold and highlighted in red represent barcoding sequence.
